# Rotenone-Induced Optic Nerve Damage and Retinal Ganglion Cell Loss in Rats

**DOI:** 10.3390/biom14091047

**Published:** 2024-08-23

**Authors:** Yasuko Yamamoto, Takazumi Taniguchi, Atsushi Shimazaki

**Affiliations:** 1 Product Development Division, Santen Pharmaceutical Co., Ltd., Nara 630-0101, Japan; 2 Ophthalmology Innovation Center, Santen Pharmaceutical Co., Ltd., Nara 630-0101, Japan

**Keywords:** mitochondrial dysfunction, rotenone, optic nerve, axon, retinal ganglion cells, intravitreal injection

## Abstract

Rotenone is a mitochondrial complex I inhibitor that causes retinal degeneration. A study of a rat model of rotenone-induced retinal degeneration suggested that this model is caused by indirect postsynaptic N-methyl-D-aspartate (NMDA) stimulation triggered by oxidative stress-mediated presynaptic intracellular calcium signaling. To elucidate the mechanisms by which rotenone causes axonal degeneration, we investigated morphological changes in optic nerves and the change in retinal ganglion cell (RGC) number in rats. Optic nerves and retinas were collected 3 and 7 days after the intravitreal injection of rotenone. The cross-sections of the optic nerves were subjected to a morphological analysis with axon quantification. The axons and somas of RGCs were analyzed immunohistochemically in retinal flatmounts. In the optic nerve, rotenone induced axonal swelling and degeneration with the incidence of reactive gliosis. Rotenone also significantly reduced axon numbers in the optic nerve. Furthermore, rotenone caused axonal thinning, fragmentation, and beading in RGCs on flatmounts and decreased the number of RGC soma. In conclusion, the intravitreal injection of rotenone in rats induced morphological abnormities with a reduced number of optic nerve axons and RGC axons when the RGC somas were degenerated. These findings help elucidate the pathogenesis of optic neuropathy induced by mitochondrial dysfunction.

## 1. Introduction

Mitochondria play an important role in respiratory cell processes, metabolism, energy production, free radical production, and apoptosis [1]. The alterations of mitochondrial function impact cellular metabolism and critically influence whole-body metabolism, health, and life span [1]. In addition, the energy supply required for neuronal survival and excitability is largely dependent on the mitochondria in the central nervous system (CNS); therefore, the neuronal tissues are much more vulnerable to mitochondrial dysfunction [2]. Mitochondrial dysfunction is involved in the pathogenesis of neurodegenerative diseases including Parkinson’s disease, amyotrophic lateral sclerosis, multiple sclerosis, glaucoma, and Leber’s hereditary optic neuropathy (LHON) [2,3].

Mitochondrial complexes (I–V) are localized in the mitochondrial inner membrane and are involved in the oxidative phosphorylation system [4]. Chemical treatments that damage mitochondrial complex I induce the specific neurodegenerative disease phenotypes in animals, probably by reducing adenosine triphosphate (ATP) synthesis and overproducing reactive oxygen species, which are the common byproducts of oxidative reactions occurring in the mitochondria [3].

Rotenone is a naturally occurring, broad-spectrum pesticide that inhibits NADH dehydrogenase in mitochondrial respiratory chain complex I. Rotenone is widely used to study mitochondrial function and oxidative stress in neuronal cell death. The induction of neurodegeneration with rotenone results in LHON phenotypes in animals [5,6]. LHON is the most common hereditary optic neuropathy, causing severe visual loss [7,8] resulting from the point mutations of mitochondrial DNA (mtDNA) in the genes encoding the subunits of mitochondrial complex I [3]. Interestingly, although these mtDNA mutations are presented throughout the cells of the body, the degeneration is manifested specifically in the retinal ganglion cells (RGCs) and the optic nerves in human LHON [7,9]. Moreover, recent studies have shown that the intravitreal injection of rotenone induces RGC damage in rodents [10,11]. Sasaoka et al. also showed that intravitreal rotenone injection downregulated the expression of neurofilament light chain (Nfl) (specifically expressed in RGCs in retinal tissues) and induced cell loss in the ganglion cell layer (GCL) in rats [12]. Rotenone also significantly reduced the number of RGCs and decreased the thickness of the retinal nerve fiber layer and GCL in addition to the reductions observed in other retinal layers, such as the inner plexiform layer and inner nuclear layer, in retinal cross-sections in mice [11].

These recent studies have further supported the validity of the rotenone-induced experimental disease model for LHON. In this optic neuropathy, the optic nerve is the primary injury site, and therefore, the study of axonal degeneration in the optic nerve is vital for understanding the disease’s pathogenesis. However, axonal degeneration (especially in the optic nerve) in LHON animal models with rotenone has not yet been fully investigated.

This study investigated the effects of an intravitreal injection of rotenone on the morphological changes in axons in the optic nerves and RGCs. We also evaluated the relationship between these changes and RGC soma damage induced by rotenone.

## 2. Materials and Methods

### 2.1. Animals

Male Sprague Dawley rats (Charles River Laboratories, Wilmington, MA, USA) weighing 190–240 g were used in this study. The animals were housed under a 12 h light–dark cycle. AAALAC International fully accredits the rodent care program, and, although not directly relevant to the studies described here, the facility is also licensed by the United States Department of Agriculture to conduct research. All the animal studies were reviewed and approved by EyeCRO’s Institutional Animal Care and Use Committee and align with the Animal Research: Reporting of In Vivo Experiments (ARRIVE) guidelines. All the procedures adhered to the Association for Research in Vision and Ophthalmology (ARVO) Statement for the Use of Animals in Ophthalmic and Vision Research, as well as state guidelines and local regulations.

### 2.2. Reagents

Rotenone (Sigma, Saint Louis, MO, USA) was prepared just before use by first dissolving an appropriate amount of rotenone into 100% dimethyl sulfoxide (DMSO) Hybri Max (Sigma, Saint Louis, MO, USA) in a tube at 5 mg/mL solution. The rotenone solution was then diluted to 50% DMSO by adding an appropriate volume of HyClone HyPure water for sterile water injection (Cytiva, Logan, UT, USA). For the vehicle preparation, DMSO was diluted to 50% DMSO by adding HyClone HyPure water.

### 2.3. Intravitreal Injection

The animals were first anesthetized with isoflurane (3.5% for induction and 2.5% for maintenance). The pupils were then dilated according to a standard procedure with 1.0% cyclopentolate and 10% phenylephrine [13]. A total volume of 5 µL per eye was injected into the vitreous at the pars plana using a blunt tip Hamilton syringe, and a 33-gauge needle was inserted into the eye from the nasal sclera at 1.5 mm posterior to the limbus, inserted toward the optic disk to a depth of 2.5 mm. Following injection, topical Terramycin ophthalmic ointment (Zoetis, Parsippany, NJ, USA) was applied to the eyes.

### 2.4. Immunohistochemistry and Quantification of the RGC Number

The animals were sedated by the intraperitoneal administration of Ketamine/Xylazine and then euthanized by the intracardial administration of Euthasol^®^ (Virbac AH, St. Louis, MO, USA). Following euthanasia, the eyes were enucleated and fixed in 4% PFA for 2 h at room temperature and stored in 1 × phosphate-buffered saline (PBS) at 4 °C until retina dissection. The fixed eyes were dissected to keep each retina protected from light throughout the staining procedure. The retinas were double-labeled with anti- RNA-binding protein with multiple splicing (RBPMS, 1:500 dilution, GeneTex, Irvine, CA, USA) and anti-beta-tubulin III (TUJ1, 1:1000 dilution, BioLegend, San Diego, CA, USA) primary antibodies, incubated in appropriate secondary antibodies (1:1000 dilution donkey anti-rabbit 555, and 1:1000 dilution donkey anti-mouse 488, ThermoFisher, Waltham, MA, USA), flatmounted onto a microscope slide, and imaged using a Nikon upright epifluorescence microscope. Eight separate 40× images located 200 µm from the outer retina were captured from the peripheral retina. The double-labeled images were analyzed using the CellProfiler software [14] (version 3.0, Broad Institute, Cambridge, MA, USA) to quantify RBPMS-positive cells.

### 2.5. Histological Evaluation of Optic Nerves and Quantification of Axon Numbers

The morphological evaluation of the optic nerves was conducted with reference to Ghnenis et al.’s method [15]. Following the euthanasia and eyeball enucleation, the optic nerves were dissected and fixed in 2% paraformaldehyde (PFA) fixative/2.5% glutaraldehyde overnight at 4 °C. The optic nerves were then transferred to 0.4% PFA and stored at 4 °C. The optic nerves were post-fixed in 2% osmium tetroxide, processed through a series of ethanol washes, and embedded in epon/Araldite resin. A 1-micron-thick transverse section was collected and counter-stained with toluidine blue, as described in Lim et al. [13]. The entire cross-section was imaged using a 100× oil immersion objective lens (MicroVisioneer Software, 2020 edition of manualWSI, Neckar, Germany). The images were post-processed for contrast enhancement, followed by quantification using the AxoNet plugin from ImageJ (version 1.52t, Wayne Rasband at the National Institutes of Health Bethesda, MD, USA), which applies a pixel-wise axon density estimation integrated over the predetermined area to calculate total surviving axon counts [16]. One section per nerve was analyzed.

### 2.6. Statistical Analyses

Statistical analyses were performed with EXSUS (v. 10.0.3) using Welch’s *t*-test. Differences were assumed to be statistically significant when *p*-value < 0.05.

## 3. Results

### 3.1. Axonal Degeneration in the Optic Nerve by Intravitreal Injection of Rotenone

**Figure 1:**
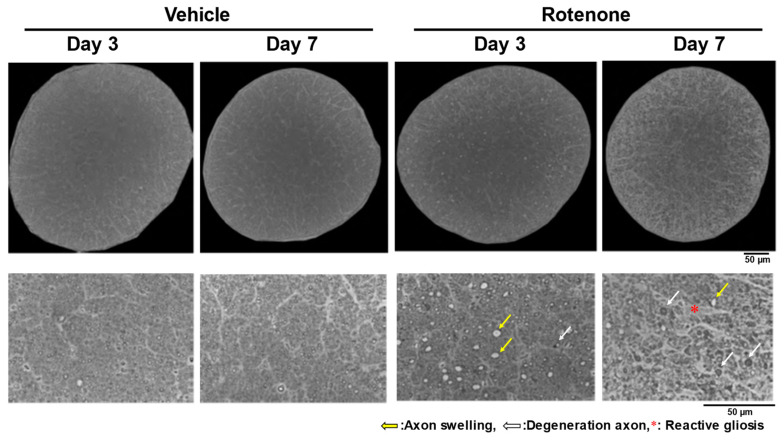
Axon swelling, axon degeneration, and reactive gliosis in the optic nerves after rotenone injection. After the intravitreal injection of rotenone, the axons in the optic nerves were morphologically evaluated on day 3 and day 7. The optic nerves were counter-stained with toluidine blue. The entire cross-section was imaged using a 100× oil immersion objective lens. The yellow arrows indicate axon swelling. The white arrows indicate degenerative axons. The red asterisk indicates reactive gliosis. Dimethyl sulfoxide was used as the vehicle control. The scale bar corresponds to 50 µm.

After the intravitreal injection of rotenone (10 nmol), axon swelling and degeneration occurred in the optic nerves on days 3 and 7, with severe morphological changes on day 7 (Figure 1). In addition, while reactive gliosis was not apparent on day 3, it was observed on day 7 (Figure 1).

**Figure 2:**
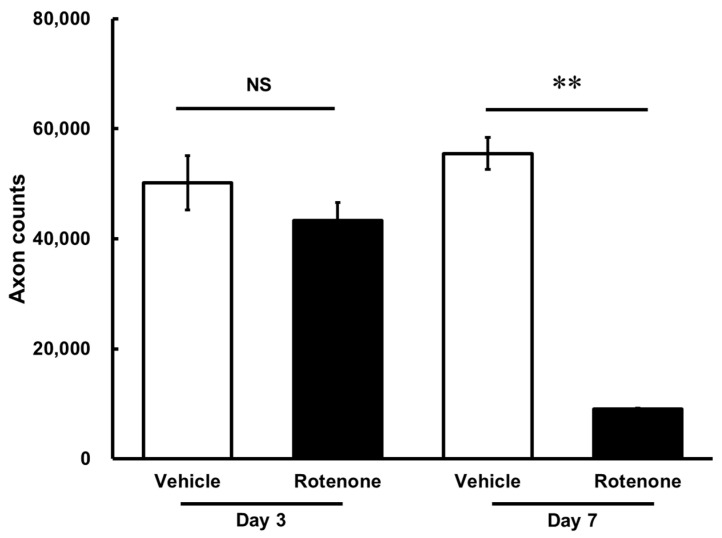
Quantification of axon numbers in the optic nerve after the rotenone injection. The axon numbers in the optic nerves were assessed using the AxoNet plugin from ImageJ on day 3 and day 7 after the rotenone injection. Each value represents the mean ± standard error of the mean (SEM) of three eyes. Three rats were used. ** *p* < 0.01, compared to the vehicle (Welch’s *t*-test). NS: not significant.

The optic nerves were processed for the measurement of axons in the vehicle and rotenone treatment groups. On day 7, there was a significant loss of axons following the rotenone injection in the optic nerves compared with the vehicle treatment (*p* < 0.01). In contrast, rotenone did not affect axon counts on day 3 (Figure 2).

The axon counts in the rotenone-treated groups comprised 86.4 ± 6.4% and 16.3 ± 0.3% of each vehicle on day 3 and day 7, respectively (Table 1).

### 3.2. Axonal Degeneration of Retinal Ganglion Cells (RGCs) in the Retina by Intravitreal Injection of Rotenone

**Figure 3:**
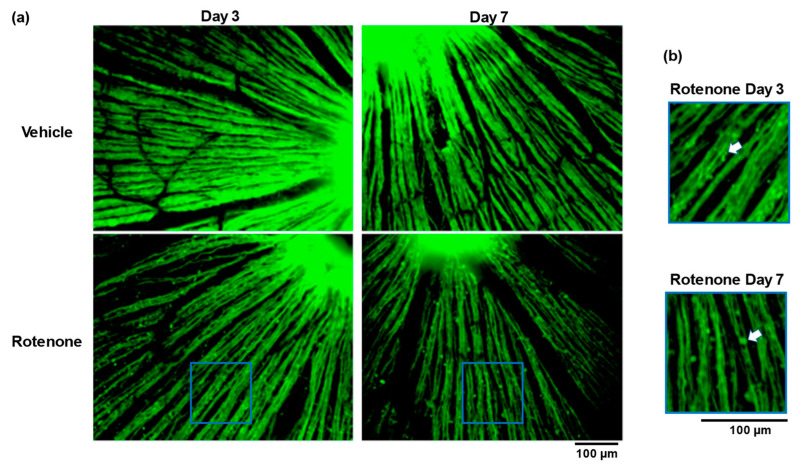
Rotenone-induced loss of axonal density and the axonal beading of RGC. The TUJ1 (axonal maker) staining of retinal flatmounts was conducted for the assessment of axon density and axonal beading following the rotenone injection at day 3 and day 7 (20× magnification) (**a**). The blue rectangle delineates the area’s higher magnification in panel a. The white arrows indicate axonal beading (**b**). The scale bar corresponds to 100 µm.

The TUJ1 staining of retinal flatmounts was conducted to evaluate the morphological changes in the RGC axons. No abnormalities appeared in the vehicle-treated eyes on day 3 or day 7. In contrast, the intravitreal injection of rotenone led to severe abnormalities, including low axonal density and fragmentation at both time points (Figure 3a). Moreover, axonal beading was observed in the RGC axons of the rotenone-treated eyes (Figure 3b).

### 3.3. Rotenone-Induced RGC Soma Damage

**Figure 4:**
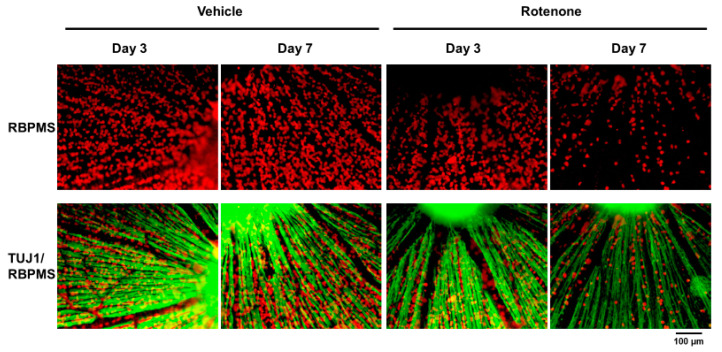
Immunohistochemistry of RGCs by RNA-binding protein with multiple splicing (RBPMS) and TUJ1 antibodies on retinal flatmounts. The double staining of RBPMS (RGC maker) and TUJ1 of RGCs was performed on retinal flatmounts following the rotenone injection on day 3 and day 7. Red represents RBPMS; green represents TUJ1 (20× magnification). The scale bar corresponds to 100 µm.

**Figure 5:**
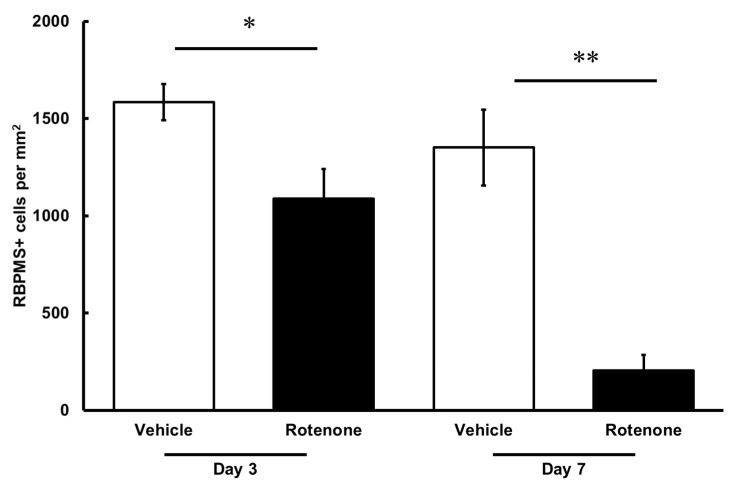
Quantification of RGCs after the rotenone injection. The RBPMS-positive cells were identified using an upright epifluorescence microscope. The CellProfiler software was used for the quantification of RGC numbers. Each value represents the mean ± SEM of 4–5 eyes. Four rats were used. * *p* < 0.05 and ** *p* < 0.01, compared to the vehicle (Welch’s *t*-test).

The RGC count was quantified by RBPMS staining of the retinas following rotenone injection (Figure 4). On both days 3 and 7, the number of RBPMS-positive cells was significantly lower in the eyes injected with rotenone than those treated with vehicle (Figure 4 and Figure 5). Relative to the vehicle, the RBPMS-positive cells comprised 68.7 ± 9.6% and 15.2 ± 5.9% of the cell counts following the rotenone injection on day 3 and day 7, respectively (Table 2).

## 4. Discussion

Our study adds to the current understanding of rotenone-induced retinal degeneration by showing that the intravitreal injection of rotenone into rat eyes (1) induced axonal swelling and degeneration, and the occurrence of reactive gliosis in the optic nerve; (2) significantly reduced axon numbers in the optic nerve; and (3) caused axonal thinning, fragmentation, and beading in RGCs on retinal flatmounts, and decreased numbers of RGC soma. Our study supports a rat model of retinal degeneration by mitochondrial dysfunction induced by the intravitreal injection of rotenone.

Mitochondrial dysfunction is one of the key factors of neurodegeneration in various CNS and ocular diseases [2]. Mitochondria generate most of the cell’s supply of ATP, used as a source of chemical energy. In particular, Na+/K+ ATPase uses half of the brain’s energy to restore the resting membrane potential in excitatory cells, requiring vast amounts of energy provided by the dense mitochondrial content of the CNS [17,18]. Accordingly, the neurodegenerative diseases of the CNS, such as Parkinson’s disease and amyotrophic lateral sclerosis, show mitochondrial dysfunction [19]. In CNS disease models, dysfunctional mitochondria reportedly contribute to axonal degeneration [20], but the involvement of mitochondrial dysfunction in axonal degeneration in optic neuropathy remains unclear. Specifically, in LHON, the optic nerve is the primary injury site; therefore, the study of axonal degeneration in LHON is vital for understanding the disease pathogenesis.

Accumulating evidence indicates that RGC damage induced by rotenone [12] is due to the enhanced production of mitochondrial reactive oxygen species leading to apoptosis [21]. Although rotenone-induced retinal degeneration is already well established [22], the axonal degeneration induced by rotenone, especially in the optic nerve of the corresponding animal models, is yet to be fully investigated. In this study, we investigated the effects of an intravitreal injection of rotenone on the morphological changes in optic nerves with RGCs in rats. By day 7, the treatment with injected intravitreal rotenone showed axonal degeneration with a significant reduction in axons in optic nerves versus the vehicle treatment. In addition, reactive gliosis, known as glial scar formation, occurred 7 days after the rotenone injection. Rotenone induced the production of inflammatory cytokines in glial cells [23,24]. Various cytokines are linked to axonal degeneration [25,26], and the effects of reactive glial cells occur after injury in the CNS, involving cell types such as reactive astrocytes, activated microglia, and infiltrating immune cells [27]. Based on our results showing reactive gliosis in axons after rotenone injection, mitochondrial dysfunction-induced reactive gliosis might contribute to axonal degeneration.

Rotenone caused axonal beading in the axon of RGC on days 3 and 7 (Figure 3). It is not known whether mitochondrial dysfunction is directly associated with axonal beading. The beading of neurites induced by the activated microglia is an early feature of neuronal dysfunction toward neuronal death due to the inhibition of mitochondrial respiration and axonal transport [28]. In this study, reactive gliosis after rotenone injection was observed (Figure 1). Although further research, such as the quantification of gliosis, is needed, mitochondrial dysfunction may be causing the activated microglia-induced axonal beading.

Furthermore, axonal swelling after the intravitreal injection of rotenone was observed. Axonal beading, or the formation of a series of swellings along the axon and their retractions, are commonly observed shape transformations that precede axonal atrophy in Alzheimer’s disease, Parkinson’s disease, and other neurodegenerative diseases [29]. This is the first reported observation of the histological abnormalities of axons in optic nerves and RGCs in an in vivo rat rotenone model. Axonal atrophy was thought to occur via damage to the soma, and axonal degradation via beading was also triggered by axonal crush or transection due to injury, a process known as Wallerian degeneration [30,31]. Our results showed that rotenone-induced mitochondrial dysfunction caused axonal swelling and beading followed by the loss of axonal counts. In addition, a previous report suggested that the axonal beading is caused by the membrane tension-driven instabilities occurring when microtubule integrity is compromised [29]. In our experiments, the loss of axonal density and beading in RGC were confirmed following the rotenone injection on day 3 and day 7. The number of axons did not decrease, whereas axon swelling and axonal degeneration were observed on day 3. Axonal injury, in the intracerebral hemorrhage model, results from microtubule disassembly and mitochondrial dysfunction [32]. Although further research, such as the quantification of axonal swelling, is needed, these observations suggest that mitochondrial dysfunction on RGC soma or axons may damage microtubules after rotenone injection as a secondary effect, leading to axon beading or swelling.

The evaluation of RGCs on flatmounts showed rotenone decreased the number of RGCs on day 3 and day 7. Our results are consistent with previous reports indicating that the intravitreal injection of rotenone significantly reduced the number of RGCs in retinal cross-sections and gene expression of Nfl in the retina [11,12].

## 5. Conclusions 

The results of this study confirmed the axonal degeneration in the optic nerve and the damage of RGC after rotenone-induced mitochondrial dysfunction via an intravitreal injection. These fundamental findings of rotenone-induced axonal degeneration in the optic nerve and RGC, potentially caused by mitochondrial dysfunction, contribute to the understanding of LHON pathogenesis and other ocular diseases involving RGC and optic nerve damage with mitochondrial dysfunction in conditions such as glaucoma.

## Figures and Tables

**Table 1:** Change in axon degeneration.

Group		Average (% of Vehicle)	SEM
Day 3	Vehicle	100	9.8
	Rotenone	86.4	6.4
Day 7	Vehicle	100	5.2
	Rotenone	16.3	0.3

Numbers represent the percentages of axon numbers relative to those in each vehicle on day 3 and day 7, respectively. Each value represents the average and SEM of 3 eyes of 3 rats.

**Table 2:** Change in RGC loss.

Group		Average (% of Vehicle)	SEM
Day 3	Vehicle	100	6
	Rotenone	68.7	9.6
Day 7	Vehicle	100	14
	Rotenone	15.2	5.9

Numbers represent the percentages of RGC counts relative to those in the vehicle controls on day 3 and day 7, respectively. Each value represents the average and SEM of 5 eyes of 4 rats.

## Data Availability

All the data generated or analyzed during this study are included in this published article.
